# Update on Disease-Modifying/Preventive Therapies in Alzheimer’s Disease

**DOI:** 10.1007/s13670-015-0141-x

**Published:** 2015-07-30

**Authors:** Jeffrey T. Apter, Kuntal Shastri, Katherine Pizano

**Affiliations:** Princeton Medical Institute, Global Clinical Trials, Global Medical Institute, LLC, Princeton University, Princeton, NJ USA; Princeton Medical Institute, Global Medical Institute, LLC, Princeton, NJ USA; Princeton Medical Institute, Princeton University, Princeton, NJ 08455 USA

**Keywords:** Alzheimer’s disease (AD), Mild cognitive impairment (MCI), Prodromal AD, Mild AD, Early-onset Alzheimer’s disease, Late-onset Alzheimer’s disease, Medical illness, Cognition, ε4 allele, Amyloid Aβ, Amyloid plaques, Tau protein

## Abstract

Alzheimer’s disease (AD) is increasingly becoming a major health problem throughout the US and Western Europe. As the remnants of the Baby Boom generation begin to reach their seniority at the turn of the twenty-first century, the disease has been unwillingly brought to the attention of the public eye. A disease that has traditionally been associated with an aging population has thus become a heated topic of discussion as modern research attempts to prevent and treat this major health burden and plague of the next decade.

## Introduction

In the field of Alzheimer’s research, there are currently two contesting debates towards the causation of this hindering disease. The first, also known as the amyloid cascade theory, correlates the onset of Alzheimer’s to the deposition of an abnormal protein called amyloid-beta (Aβ) and stands as the most studied explanation of AD [1]. Those who support this viewpoint have been known as “baptists.”

The second competing hypothesis, which is slowly gaining more attention, associates the onset of Alzheimer’s disease instead to fibrillary tangles called tau protein. Scientists in support of this theory, the “tauists,” are attempting to target and block tau hyperphosphorylation in Alzheimer’s patients in hopes of relieving the negative consequences of the disease.

New innovations, taking into account both perspectives, are changing the field of Alzheimer’s research. Whereas before scientific endeavors sought to target the disease in its later stages, now a more preventative approach is being undertaken in hopes of stopping the onset of the disease altogether. In support of either theory of AD, there is an overwhelming consensus that Alzheimer’s disease can exist in two distinct forms. These dichotomous variants consist of the more common late-onset Alzheimer’s (LOAD) and a more genetic form called early-onset Alzheimer’s (EOAD). Current research seeks to target either form in its earliest stages, even before the very first symptomatic signs of deterioration appear [2].

The major risks for developing LOAD are the APOE ε4 gene and aging. Individuals carrying both alleles of this gene, as opposed to those who have the ε3 alleles, have a 50 % greater risk of developing the disease [3, 4]. Researchers are also aware that individuals carrying both ε2 alleles have a 50 % reduced risk of ever contracting Alzheimer’s [3, 4]. There are, however, other non-genetic predispositions towards the development of AD. These risk factors include various environmental or epigenetic stimulants like decreased hippocampal volume, being amyloid positive, or having a CSF marker for AD such as low CSF Aβ and increased/phosphorylated tau protein [2]. We also now recognize that there may be several stages of the disease even before the development of AD is evident. Amyloid plaques may be seen in the brains of 20–30 % of normal older individuals through PET imaging and yet it may take years between an abnormal test and the development of AD [5]. Evidence such as this confirms the existence of a pre-clinical form of AD called mild cognitive impairment (MCI). Slowly, the analysis of these abnormal CSF levels along with the evolving use of PET imaging scans to detect amyloid plaques are beginning to constitute the bio-markers for the clinical diagnosis of MCI due to AD and thus helping to provide the answers needed to halt the onset of dementia in patients.

## Current Research

Although medical treatment will be an important component of AD management in the future, there are several encouraging trends towards early preventative measures to reduce the incidence of AD. Simple lifestyle changes such as 20 min of daily exercise, a Mediterranean diet, and early diagnosis and treatment of diabetes concurrent with low cholesterol levels all help decrease the risk of AD. Keeping the brain mentally active with tasks such as learning new languages, increased social interaction, and other complex learning processes have also been found to be essential remedies, which could aid in the prevention of Alzheimer’s disease.

However, these preemptive strategies are not always enough, especially for individuals who are genetically predisposed to Alzheimer’s. Here, medical innovations must take the lead instead, in hopes of relieving the strain the disease causes to both the patient and their family members. Yet although many drugs are currently in the pipeline for AD, no drugs have been successfully approved in the EU and the USA in almost 15 years. Current treatments available for Alzheimer’s disease in the market consist mostly of cholinesterase inhibitors such as donepezil, galantamine, and rivastigmine as well as the NMDA receptor uncompetitive antagonist memantine [2]. These drugs, which are commonly referred to as symptomatic treatments, are only helpful for a limited time span in improving the cognitive abilities of patients with AD. Consequently, none of these drugs offer the long-term answers we are currently seeking: finding true disease modifiers. In light of this, the research being done on the formation of amyloid β and/or neurofibrillary tangles of tau protein aggregates is essential for finding a more stable, long-term answer for the longevity as well as mental and functional capabilities of individuals afflicted with Alzheimer’s disease. In the near future, this research will provide the answer to the cure and possible early prevention of Alzheimer’s disease.

## The Baptists’ Perspective

### The Inhibitors

The amyloid cascade theory has launched several promising studies in the field of Alzheimer’s research, focusing on the specific targeting of the formation and accumulation of Aβ into amyloid plaques. The production of amyloid β plaques in patients with Alzheimer’s involves two essential steps. The first step comprises of the cleavage of the amyloid precursor protein (APP) by the beta-site amyloid precursor protein-cleaving enzyme (BACE1/β-secretase), an enzyme found only in Alzheimer’s patients, producing the C99 fragment [6]. Concurrently, the next step involves the cleavage of this C99 compound by *γ*-secretase, which leads to the production of amyloid oligomers. The clumping together of these amyloid β oligomers to form amyloid plaques can then prevent cellular signaling at neuron synapses, causing AD. It is important to note that in healthy individuals, the ∝–secretase enzyme is used to cut APP instead of BACE1, suggesting a strong correlation between elevated levels of the BACE1 and late-onset Alzheimer’s in patients. To this end, an array of novel strategies have begun to target directly/indirectly the accumulation of Aβ deposits through the use of monoclonal antibodies, antigens, beta and gamma secretase inhibitors, and anti-amyloid aggregation compounds. The inhibition of *γ*-secretase or BACE1, two mechanisms essential to the amyloid cascade theory, would thus, in theory, indirectly prevent amyloid plaque buildup.

Among the new BACE1 inhibitors under active development are the small molecules MK-8931 (Merck), AZD3293 (AstraZeneca/Lilly), E2609 (Eisai/Biogen), and JNJ 54869111 (Janssen). These new compounds are looking to find the answers that their predecessors could not, since the several gamma secretase inhibitors such as semagacestat (Lilly), begacestat (Pfizer), and avagacestat (Bristol-Myers Squibb) all failed in clinical studies and have thus been discontinued.

### MK-8931 (Merck)

MK-8931, another BACE1 inhibitor [7•], showed promising phase 1 results and is now currently enrolling participants in their P3 trials to test the effect of the molecule on a larger sample size.

### AZD3293 (AstraZeneca/Lilly)

This BACE1 inhibitor is in a late-stage BACE1 program that has produced good CSF AB reduction in P1. The collaborative effort of both AstraZeneca and Lilly wishes to target and prevent the cleavage of the APP protein and subsequent release of the C99 fragment by β-secretase. Both companies are looking to combine the phase 2/3 trials for the drug, having a large joint phase 2/3 trial called AMARANTH with over 1000 patients with MCI due to AD or mild AD [8].

### JNJ 54869111 (Janssen)

Presently, there are several more studies with promising results underway that may alter the treatment of AD in the future. Among the BACE1 secretase inhibitors, JNJ 54869111 has shown outstanding results as a potent brain penetrant achieving up to 95 % Aβ reduction with once daily oral dose [5]. Developed by Shionogi in October 2012, the drug hopes to investigate certain key features such as subject engagement and responsiveness, and the effects, safety, and tolerability of multiple dosing [5]. JNJ 54869111 entered its first human dosing on April 2013 as Shionogi aims to monitor the CSF Aβ output after a variety of dosing regiments.

### PQ912 (Probiodrug)

This is a small molecule glutaminyl cyclase (QC) inhibitor. QC catalyzes the formation of the pyroglutamate-modified amyloid peptides [9], a phenomenon most common in AD patients. Probiodrug’s PQ912 drug is testing a novel implementation of QC inhibition as an indirect method of Alzheimer’s prevention.

### CAD106 (Novartis)

Novartis is taking a slightly different approach than its competitors as it plans to commence a trial of CAD106, an active immunotherapy, and the BACE1 inhibitor NB360 in up to 1300 cognitively healthy ApoE4-positive subjects [10•]. The purpose of the trial will be to determine whether the prevention of amyloid plaque is possible, the source baptists believe to be the cause of Alzheimer’s disease. The trial, which will start enrollment for the CAD106 dosing arm in late 2015, will be a decisive turning point in pin-pointing the onset of AD and could possibly shift the approaches being used in Alzheimer’s research in favor of tau inhibition mechanisms if the trials end in failure. Along with CAD106, Novartis and its partner Banner Alzheimer’s Institute will commence enrollment for the NB360 dosing arm of the study in 2016 [11••]. The implementation of NB360 in the near future seems bright as clinical trials begin after its success in reducing amyloid plaques in animal models [12•].

### Monoclonal Antibodies in Action

The potential for the treatment of AD through the use of monoclonal antibodies is also encouraging. These studies use a much more direct approach, as monoclonal antibodies bind directly to amyloid protein to prevent plaque buildup. Preclinical trials in transgenic mice have shown that implementation of N-terminus-directed antibodies against Aβ are able to effectively reduce the collection of amyloid deposits in the brain [13, 14] With this breakthrough, several monoclonal antibodies have begun to be tested in clinical trials such as bapineuzumab, gantenerumab, crenezumab, solanezumab, BAN2401, and aducanumab. Many of these new studies are focused in targeting early prevention in individuals at risk of AD due to familial and genetic factors. These studies are thus attempting to use the lag between the early formation of amyloid plaques and clinically diagnosed MCI, to attack the early onset of the disease preemptively.

### Bapineuzumab (Pfizer/Janssen Alzheimer Immunotherapy)

A humanized monoclonal antibody that is capable of binding to both soluble and fibrillar forms of Aβ [2]. Unfortunately, the clinical trials involving the phase 3 of the drug ended in disappointment, as the drug failed to show any efficacy in improving the cognitive abilities of patients afflicted with mild to moderate Alzheimer’s. Nevertheless, the connection between bapineuzumab and the lowering of p-tau, a CSF marker of neurological degradation, in both APOE ε4 gene individuals and those without the gene, provided an encouraging start to new research involving MCI due to prodromal/mild AD [2].

### Gantenerumab (Roche)

Following the failure and early termination of the gantenerumab phase 3 SCarlet RoAD trial in prodromal AD, Roche has decided to continue the P3 Marguerite Road trial for mild AD. The SCarlet RoAD trial had originally terminated after a failed futility analysis, which evaluated half (*n* = 400) of the trial population after 2 years on treatment [15•]. The P3 Marguerite Road trial shows promising results with some considerations at increasing the dosage of participants.

### Crenezumab (Roche/Genentech)

Beginning in late February 2015, Roche initiated another Alzheimer’s trial with the drug crenezumab, another monoclonal antibody targeting amyloid plaque buildup [16]. A small P1b trial of the Crenezumab IV formulation was underway with the aim to determine whether (and to what extent) the IV dose can be increased for potential evaluation in future P2 or P3 trials. Recently, Roche has launched the phase 2 trials, with participants receiving 15 mg/kg per month of the drug [16]. The phase 2 trials, targeting patients with mild to moderate Alzheimer’s, are expected to be ongoing until the year 2020 [16].

### Solanezumab (Lilly)

Another study is taking a novel approach by testing clinically normal individuals who have beta amyloid PET scans in a randomized clinical trial in conjunction with the Eli Lilly monoclonal antibody solanezumab [17]. Solanezumab had previously launched two phase 3 trials (EXPEDITION 1 and EXPEDITION 2) [18]. However, the drug failed to improve overall cognitive and memory abilities in patients significant slowing of cognitive decline; the pre-specified analysis of patients with mild AD did show a slight signal of improvement. Hence, Lilly initiated EXPEDITION 3 in a mild AD only population. This ongoing trial, which just completed enrollment in April/May 2015, involves participants who are all amyloid positive on a florbetapir PET scan; in theory, improving the likelihood of success for solanezumab in EXPEDITION 3.

### BAN2401 (Eisai/BioArctic/Biogen)

Eisai and BioArctic’s BAN2401 drug is currently undergoing phase 2 clinical trials, after a successful phase 1 run [19••]. Many are hopeful for the future indications of this drug, which can serve as a selective immunotherapy technique against AD if it achieves to successfully bind and neutralize the neurodegenerative amyloid protein formation found in patients with dementia. Even more exciting is Eisai’s employment of the innovative Bayesian trial. The Bayesian design will allow for the potential early termination of the P2 trials before full enrollment if either of two criteria are met: strong efficacy signals of the drug (allowing advancement to P3) or interim futility (allowing discontinuation of the program and thus reduction in R&D investment) [20].

### Aducanumab (Biogen)

Aducanumab, an anti beta-amyloid monoclonal antibody, is yet another study being conducted in subjects with prodromal or mild AD. At the recent ADPD 2015 conference in Nice, Biogen Idec presented a positive phase 1a study with a statistically significant reduction of amyloid plaques as measured by PET imaging after 1 year as well as a significant decline on Clinical Dementia Rating Sum of Boxes (CDR-SB) in the first year of treatment. Some of these findings helped to renew optimism for the amyloid hypothesis, which was losing acceptance after several years of negative results. There was also a high incidence of amyloid-related imaging abnormalities/microhemorrhages (ARIA-E), which was dose dependent and more common in the ApoE4+ subjects [5]. ARIA tended to start early, was generally mild and transient, and a majority of patients continued treatment with a lower dose of aducanumab (BIIB-037).

### DIAN Initiative

An initiative called DIAN (Dominantly Inherited Alzheimer Network) is conducting a multitude of studies on families with dominant genetic mutations [21]. The DIAN studies show an international effort of attempting to make future AD medication accessible for persons of all nationalities.

### Alzheimer’s Prevention Initiative (API)

Another study led by the Alzheimer’s Prevention Initiative has been launched with a focused attention to a section of the Colombian population. These trials involve Colombian families with Basque ancestry from the Antioquia region, like the Cuartas family, which have developed cases of EOAD [22, 23]. These Colombian families are affected by a presenilin I genetic mutation which will cause the development of Alzheimer’s disease to all the offspring which inherit this mutated gene.

### ADCS-A4 trial

Yet another collaborative effort in the field of Alzheimer’s, the A4 trials are focusing their efforts at analyzing the effect solenezumab will have on 1150 adults who are all amyloid positive but have yet to show signs of cognitive impairment [24•]. The project, which began in 2014, is currently running phase III trials and looking forward to a possible 2-year extension in the near future [23].

### TOMORROW Trial

Along with the previous three programs mentioned above (DIAN, API, ADCS-A4), the TOMORROW trials represent the Collaboration for Alzheimer’s Prevention (CAP). The TOMORROW trial is researching the effects that low-dose pioglitazone will have in patients with the TOMM40 genetic mutation, a mutation that heightens the possibility for neurogenesis and the onset of MCI due to AD [25].

## Limitations and Future Directions of Amyloid PET in Clinical Practice

Amyloid imaging with PET scans has allowed for more confident diagnosis of AD and is being used much more frequently in clinical trials. The PET imaging scans can easily detect the presence of Aβ in the brain. Currently, most of the studies involving the amyloid cascade theory make strategic use of PET scans in the determination of possible research participants. These amyloid PET scans have thus become a recent commodity in the diagnosis of possible AD onset in research subjects. Following the success of this early diagnosis, tau tracers are also currently under development. However, while amyloid PET scans are becoming an invaluable research and diagnosis tool, several improvements and impediments still exist. While the beneficial outcomes of PET have been a clear-cut distinction in clinical trials, US Medicare does not yet cover its full cost of $3500 for diagnostic practices [5]. If amyloid PET scans become more common in the diagnosis of AD in patients not participating in clinical trials, then they need to become more cost effective as well. Moreover, the implementation of more quantitative measures would be a strong asset as the PET scans currently only indicate if an individual is amyloid positive or negative. More prognostic data, such as a percentage measurement of an individual’s amyloid plaque burden, can be a helpful tool in future research initiatives. However, the biggest impediment with the success of amyloid PET scans seems to be the lack of effective therapeutic intervention. Even if patients test amyloid positive, we currently lack the instrumentations necessary to implement the appropriate follow-up treatments to stop the neurodegenerative progression of the disease.

## The Tauist Outlook

Hyperphosphorylation of a microtubule-associated protein known as tau leads to the formation of neurofibrillary tangles in neurons. Neurofibrillary tangles aggregate, or group, in an insoluble form in neurons affecting normal neuronal functions. Several molecules are involved in the phosphorylation of tau including the kinases namely the glycogen synthase kinases (GSK-3) and cyclin-dependent protein kinase (CDK). While studies involving these molecules are further behind those that target the amyloid cascade theory, there have been some promising starts with compounds aimed at inhibiting the tau pathways in novel ways. Several compounds have been developed such as valproate, lithium, and Methylene blue [26]. Other innovative efforts have also begun in the development of anti-tau vaccines.

### Methylene Blue in Perspective

Of all the tau targeting compounds, Methylene blue seems to have the most promising results. The compound, which is a type of phenothiazine, has been shown to reduce tau aggression in transgenic mice. The trial, using P301L tau transgenic mice, gave several oral administrations of Methylene blue for 5 months to test the effects of the compound in vivo [27]. The results were successful, depicting a tau aggression decrease in the mice, giving the scientific community high hopes of using the compound in future human trials for the possible treatment of AD via anti-tau phosphorylation. With these results in mind, TauRx Pharmaceuticals recently announced the completion of their phase II clinical trials involving the testing of this Methylene Blue compound in a sample size of 321 adults afflicted with mild/moderate AD [28].

### ACI-35 (AC Immune/Janssen)

ACI-35 is one of the first drugs of its kind. Developed through the AC Immune program, this compound is a pioneer anti-tau vaccine currently in phase 1b. Researchers hope the vaccine will help stimulate the patients’ immune systems against misfolded and phosphorylated tau proteins involved in the onset of Alzheimer’s [29].

### AADvac1 (AXON Neuroscience)

In 2013, AXON Neuroscience’s AADvac1 began its first P1 trials for another vaccine targeting anti-tau phosphorylation. AADvac1 consists of synthetic polypeptides, which are meant to elicit an immune response against pathogenic tau proteins [30•].

## Conclusion

Moving forward, we recognize that, potentially, single agents will not be effective in the treatment of Alzheimer’s disease. Instead, the answers sought in this multifactorial disease involving multiple pathways and pathologies can be found in a combination of clinical methodologies and discoveries. The more we fail at single protein-targeted therapies, the more we lean towards a belief that AD is a heterogenic, complex disorder with deregulation of multiple pathways due to genetic, epigenetic, and environmental reasons. Hence, AD needs to be approached using a systems biology/genomic/transcriptomic/proteomic method to get a more comprehensive picture of the cause, effect, and treatment options. Combinational therapies targeting both Aβ accumulation and tau protein malformation may just be the answer so sought after for AD research. Already, PET imaging has given both researchers and physicians a more viable way towards the diagnosis of Alzheimer’s. As future studies are concluded and more information is gathered, the efficacy of Alzheimer’s treatment will be greatly improved.

The epidemic of AD is near at hand. Unfortunately, to date, no new compounds have achieved FDA or EMA approval in the last decade and a half. The stakes are high for a drug to cross this barrier. Several hundred compounds are now under development and almost 200 trials on the way. The scientific and pharmaceutical industries are aligned with millions of dollars being poured into AD research. The scientific knowledge has made significant progress and we may look forward to breakthroughs in the next decade.
